# Preparation and In Vitro Evaluation of Alginate Microparticles Containing Amphotericin B for the Treatment of *Candida* Infections

**DOI:** 10.1155/2020/2514387

**Published:** 2020-08-01

**Authors:** Merlis P. Alvarez-Berrios, Lisa M. Aponte-Reyes, Lourdes Diaz-Figueroa, Juan Vivero-Escoto, Alexis Johnston, David Sanchez-Rodriguez

**Affiliations:** ^1^Department of Science and Technology, Inter American University of Puerto Rico, Ponce Campus, Ponce, PR, USA; ^2^Department of Chemistry, University of North Carolina at Charlotte, Charlotte, NC, USA

## Abstract

Invasive candidiasis (IC) remains as a major cause of morbidity and mortality in critically ill patients. Amphotericin B (AmB) is one of the most effective antifungal agents commonly used to treat this infection. However, it induces severe side effects such as nephrotoxicity, cardiac alterations, nausea, fever, and liver damage. The utilization of drug delivery systems has been explored to overcome these limitations. Several AmB lipid formulations have been developed and are currently available in the market. Although they have the ability to reduce the main side effects of free AmB, their high cost, necessity of repeated intravenous injections for successful treatment, and incidence of pulmonary toxicity have limited their use. In the last decades, alginate has gained significant interest in drug delivery applications as a cost-effective strategy to improve the safety and therapeutic effect of toxic drugs. In this work, the clinically relevant drug AmB was encapsulated into alginate microparticles using the emulsification/external gelation method. We hypothesize that this synthesis strategy may positively impact the antifungal efficacy of AmB-loaded MCPs toward *Candida albicans* cells while reducing the toxicity in human lung cells. To prove this hypothesis, the ability of the microplatform to disrupt the cellular membrane potential was tested and its antifungal effectiveness toward *Candida albicans* cells was evaluated using the cell counting and plate count methods. Moreover, the toxicity of the microplatform in human lung cells was evaluated using CellTiter 96® AQueous cell viability assay and qualitative diffusion analysis of acridine orange. Our results demonstrated that the platform developed in this work was able to induce antifungal toxicity against *Candida albicans* yeast cells at the same level of free AmB with minimal toxicity to lung cells, which is one of the main side effects induced by commercial drug delivery systems containing AmB. Overall, our data provides convincing evidence about the effectiveness of the alginate-based microplatform toward *Candida albicans* cells. In addition, this vehicle may not require several infusions for a successful treatment while reducing the pulmonary toxic effect induced by commercial lipid formulations.

## 1. Introduction

Invasive candidiasis (IC) is a serious infection caused by a yeast called *Candida*, with candidaemia being its most common clinical presentation [1–5]. One of the species responsible for this disease is *Candida albicans* which accounts for over 50% of all cases [6–8]. IC has been a major cause of morbidity and mortality in critically ill patients [1, 6, 9]. Particularly, patients that have HIV or diabetes mellitus or are undergoing a treatment that affects their immune system such as organ transplantation or chemotherapy are prone to obtain this type of infection [10]. IC affects more than 250,000 people every year and the mortality rate ranges from 40 to 70% [8, 11–16]. According to the Centers for Disease Control and Prevention (CDC), it is expected that approximately 25,000 cases of healthcare-associated invasive candidiasis occur each year in the United States [1, 17].

One of the first-line therapies used to fight this infection is the polyene macrolide antibiotic amphotericin B (AmB) [2, 3, 18, 19]. Its mechanism of action is based on the binding of the hydrophobic moiety of the molecule to the ergosterol of the fungal cell membrane, which disrupts the cellular membrane and causes the cytoplasmic content to leak out leading to cell death [20, 21]. Unfortunately, this antimycotic drug induces severe side effects such as nephrotoxicity, cardiac alterations, nausea, fever, and liver damage [3, 22, 23]. The encapsulation of AmB into drug delivery systems has been explored as an alternative to improve its tolerability [2, 3, 24]. Currently, several lipid-based formulations such as the lipid complex Abelcet® and the liposomal product Ambisome^®^ have been developed and are available in the market [25, 26]. Although these lipid-based formulations reduce the main side effects of AmB, their high cost, high-dose requirements, necessity of repeated intravenous injections for successful treatment, and incidence of pulmonary toxicity have limited their use [25–33]. Therefore, extensive research has been done to develop new low-cost approaches to improve the safety and therapeutic effect of this drug [3, 25, 26, 34, 35]. For example, AmB was encapsulated into PLGA nanoparticles. This vehicle showed lower toxicity when compared to free AmB and the *in vitro* antifungal effectivity was the same as that induced by the free drug [3]. On the other hand, the utilization of PLGA based fibrin microspheres as a delivery vehicle for AmB demonstrated that this platform was more effective in avoiding side effects when compared to the free drug and its antifungal efficacy was superior when compared to the free AmB [35]. In the last decades, alginate-based carriers have gained significant interest [34]. It is a naturally occurring polymer which is known to be biodegradable and biocompatible [36–38]. Several studies have developed alginate-based vehicles as carriers for various toxic drugs [34, 36, 39–41]. Nevertheless, only a few studies have fabricated alginate-based vehicles using different methods for the delivery of AmB [42, 43]. Sangeetha et al. formed a polyelectrolyte complex by mixing alginate and poly-L-lysine and encapsulated AmB into alginate/poly-L-lysine nanoparticles. The polyelectrolyte complex was formed to induce a slow and sustained release of the drug. The AmB-loaded alginate/poly-L-lysine nanospheres were less toxic than the free drug. The authors suggested that the nanovehicle containing AmB was mainly accumulated in the lungs and liver, while the principal side effect of AmB was decreased due to the reduced uptake of the nanoparticles in the kidneys [43]. The *in vitro* antifungal toxicity was not performed. Another strategy to decrease the side effects of AmB is the synthesis of alginate-AmB conjugates [42]. Ravichandran and Jayakrishnan obtained AmB conjugates by reacting AmB with sodium alginate via imine and amine linkages. The conjugates were encapsulated into gels discs of gelatin cross-linked with oxidized alginate. Although these conjugates were not toxic to human mammalian cells, the antifungal efficacy against *Candida albicans* yeast cells was less than that of the free drug. This may be attributed to the slow release of the drug. It was reported that the drug released from the gel discs was approximately 15–20% in 2 days [42]. Based on these results, alginate-based vehicles may confront similar clinical limitations as the commercial lipid formulations, requiring repeated intravenous injections for successful treatment and inducing pulmonary toxicity.

In this study, we encapsulated the clinically relevant drug AmB into alginate microparticles using the emulsification/external gelation method. It has been reported that the production methods of alginate nano- or microparticles have a significant impact on tuning the (bio)pharmaceutical performance [44]. We hypothesize that this synthesis strategy may positively impact the antifungal efficacy of AmB-loaded MCPs toward *Candida albicans* cells while reducing the toxicity in human lung cells. To prove this hypothesis, the *in vitro* therapeutic efficacy of the microsystem toward *Candida albicans* was evaluated using the cell counting and plate count methods and through membrane depolarization studies using bis-(1,3-dibarbituric acid)-trimethine oxonol [DiBAC_4_(3)]. Moreover, the toxicity of the microplatform in human lung cells was evaluated using CellTiter 96® AQueous cell viability assay and qualitative diffusion analysis of acridine orange. To our knowledge, no study has been carried out to study the *in vitro* effectiveness and toxicity of alginate microparticles containing AmB using emulsification/external gelation method as described in this work.

Our results demonstrated that AmB-loaded MCPs were able to disrupt the cellular membrane potential of *Candida albicans* yeast cells and the *in vitro* antifungal effectivity was the same as that induced by the free drug. Moreover, the microvehicle showed minimal lung cell toxicity and cell membrane damage. Overall, this data provides convincing evidence of the effectiveness of the platform developed in this work toward *Candida albicans* cells, which in turn may reduce the side effect of current vehicles associated with pulmonary toxicity.

## 2. Materials and Methods

### 2.1. Materials

Low viscosity alginic acid sodium salt, calcium chloride, amphotericin B, isopropanol, span 80, bis-(1,3-dibutylbarbituric acid) trimethine oxonol (DiBAC_4_(3)), and paraffin oil were obtained from Sigma-Aldrich (St. Louis, MO, USA) and were used as received without further purification. Roswell Park Memorial Institute (RPMI 1640), penicillin-streptomycin, phosphate buffer saline (PBS, 1X), and trypsin were purchased from Corning. Fetal bovine serum (FBS) was purchased from Atlanta Biologicals. CellTiter 96® AQueous Assay was obtained from Promega (Madison, WI, USA). Acridine orange solution was purchased from Invitrogen.

A Multiskan FC plate reader by Fisher Scientific was used for the cell viability analysis. An Olympus Fluoview FV 1000 confocal fluorescence microscope system was used for the qualitative diffusion analysis experiments.

### 2.2. Synthesis and Characterization of AmB-Loaded MCPs

AmB-loaded MCPs were fabricated using an emulsification method previously reported in the literature with some modifications [45]. Briefly, 10 mg of AmB was dissolved in 10 mL of an aqueous solution of alginate (5% wt/vol). Then, the alginate solution containing AmB was dispersed into a mixture of paraffin oil and span 80 (40 mL of paraffin oil and 2 mL of spam 80) at 1200 rpm for 30 minutes. Afterward, 20 mL of a 10% CaCl_2_ solution was added and stirred at 800 rpm for an additional 2 hours. After this period of time, 20 mL of isopropanol was added and the mixture was allowed to react for 10 minutes. The final product was separated by centrifugation, washed three times with isopropanol, let dry at room temperature, and stored at −20^o^C. AmB-loaded MCPs were characterized using different techniques: the hydrodynamic diameter and *ζ*-potential were determined using a particle size analyzer (Nanoplus HD, Particulate Systems, GA, USA), particle size and morphology were estimated using an optical microscope (Nikon, Nikon Instruments, NY, USA) and field-emission scanning electron microscopy (SEM; Raith150, Raith, Dortmund, Germany), and the FTIR spectrum and the absorption spectra were determined using an IR spectrophotometer (Spectrum Two, Perkin Elmer, CT, USA) and UV-Vis spectrophotometer (Evolution 260 Bio, Thermo Fisher Scientific, MA, USA), respectively.

### 2.3. AmB Content and Encapsulation Efficiency

The amount of AmB encapsulated into alginate microparticles was determined by dissolving 10 mg of AmB-loaded MCPs in 3 mL of PBS (pH 7.4) under stirring conditions for 24 hours. After this period of time, the sample was centrifuged and the supernatant was analyzed using a spectrophotometer at a wavelength of 345 nm [43, 46]. The concentration of AmB was determined using a calibration curve from 4 *μ*g/mL to 65 *μ*g/mL of AmB. 345 nm is the wavelength at which AmB in aqueous solution shows its maximum absorbance [47]. AmB content was established as the amount of AmB (*μ*g) per mg of microparticles. The percentage of encapsulation was calculated by dividing the amount of AmB encapsulated by the initial amount of AmB added to the alginate solution. The experiment was carried out in triplicate.

### 2.4. Depolarization Studies

It is known that damage induced to cellular membranes may cause the dissipation of the membrane potential [48, 49]. Depolarization studies were performed to evaluate the ability of AmB-loaded MCPs to disrupt the cellular membrane of *Candida albicans* cells. For this experiment, the membrane potential-sensitive probe bis-(1,3-dibutylbarbituric acid) trimethine oxonol (DiBAC_4_(3)) was used. DiBAC_4_(3) is an anionic dye which only fluoresces in the microenvironment of the membrane. Normal cells have a negative internal charge and exclude the dye. When cells are exposed to AmB, the drug disrupts the membrane allowing the dye to enter and fluoresce [48, 49]. An increase in fluorescence after adding AmB indicates its ability to disrupt the cellular membrane. To carry out the assay, *Candida albicans* cells purchased from ATCC (ATCC 10231) were cultured in yeast extract peptone dextrose (YPD) agar at 37°C for 24 hours. Then, approximately four colonies were resuspended in sterile saline water, the concentration was determined using a hemocytometer, and 2 × 10^6^ cells were resuspended in 2.5 mL of sterile saline water. The fluorescence of the cell suspension was followed up for 1-2 minutes to obtain a baseline. Afterward, DiBAC_4_(3) was added at a final concentration of 22.5 *μ*g/ml and the fluorescence was monitored for 90 seconds until a stable signal was obtained. Then, free AmB or AmB-loaded MCPs were added and changes in the membrane potential were monitored. The fluorescence for this experiment was determined using a spectrofluorometer at an excitation wavelength of 490 nm. The measurements were repeated two times.

### 2.5. In Vitro Release Assay

To verify that AmB is able to be released from the microparticles, 10 mg of AmB-loaded MCPs was suspended in 2 mL of PBS under stirring at 200 rpm. At specific time intervals, 100 *μ*L of the sample was withdrawn and centrifuged and the supernatant was collected and analyzed spectrophotometrically at 345 nm. The experiment was carried out in triplicate.

### 2.6. Antifungal Activity of AmB-Loaded MCPs against *Candida albicans* Cells

The effectiveness of the platform developed in this work against *Candida albicans* cells was evaluated using the cell counting method and plate count method. To compare the effect of free AmB and AmB-loaded MCPs at the same conditions, the amount of AmB-loaded MCPs added corresponded to an equivalent amount of free AmB.

*Candida albicans* cells were obtained from American Type Culture Collection (ATCC 10231) and grown on YPD agar plates (1% yeast extract, 2% peptone, 2% dextrose, and 2% bacteriological agar) at 35°C for 24 hours. Then, approximately two colonies were transferred to a tube containing 10 mL of YPD medium and homogenized. The concentration of the cell suspension was determined using a hemocytometer and 5 × 10^3^ cells were added to tubes containing different concentrations of AmB (0.5 *μ*g/mL or 4 *μ*g/mL). The concentrations of AmB used for this study were chosen in accordance with clinical concentrations reported in the literature [50, 51]. The sterile test tubes were incubated for 48 hours at 35°C and 250 rpm. After the treatment, the viability ratio was determined by cell counting using a hemocytometer. For the plate count method, 2.5 × 10^2^ cells were spread on YPD agar plates and incubated at 35°C for 48 hours. The number of viable cells was counted and expressed in CFU/mL. The cytotoxic effect of blank microparticles (MCPs) was evaluated at the concentration of microparticles corresponding to 0.5 *μ*g/mL and 4 *μ*g/mL of AmB. The experiment was performed in duplicate.

### 2.7. Cell Culture

Because the majority of the studies related to the fabrication of polymeric vehicles have focused on evaluating their *in vitro* toxicity in human kidney cells, demonstrating their ability to significantly decrease the main side effects of AmB [25, 26, 42, 52, 53]; this study will only focus on lung cell toxicity which is one of the main side effects related to commercial formulations containing AmB. Immortalized or cancer cells lines are widely used for *in vitro* toxicology studies [54]. H-460, a human large cell lung cancer, was purchased from American Type Culture Collection (ATCC). H-460 cells were cultured in RPMI 1640 medium supplemented with 10% FBS and 1% pen-step at 37°C with 5% CO_2_ atmosphere. The culture media were changed every other day. All cell cultures were maintained in 25 cm^2^ or 75 cm^2^ cell culture flasks and the cells were passaged at 70–80% confluency every 2–4 days.

### 2.8. In Vitro Cytotoxicity

For this study, H-460 cells were seeded in a 96-well plate at a density of 1,000 cells per well in 100 *µ*L of complete media. The cells were incubated at 37°C with 5% CO_2_ atmosphere for 48 h. After removing the cell culture medium, AmB at different concentrations (10, 30, 50, and 100 *µ*g/mL) was added to the cells. To compare the effect of free AmB and AmB-loaded MCPs at the same conditions, the amount of AmB-loaded MCPs added corresponded to an equivalent amount of free AmB. Blank MCPs were used as a control at the concentrations used for AmB-loaded MCPs. After 48 h of incubation in the presence of these materials, the culture media were removed and fresh media (100 *µ*L) together with 20 *µ*L of CellTiter 96® Aqueous solution were added. The cells were incubated for 2–3 h at 37°C with 5% CO_2_ atmosphere. Cell viability (%) was calculated as follows: viability = (*A*_sample_/*A*_control_) × 100%, where *A*_sample_ and *A*_control_ denote absorbance values of the sample and control wells measured at 490 nm, respectively. The results are reported as the average ± SE of four experiments.

### 2.9. Confocal Microscopy

H-460 cells were seeded at a concentration of 5 × 10^4^ cells per well on a glass cover slip in a 6-well plate with 2 mL of complete media and incubated for 24 h at 37°C and 5% CO_2_ atmosphere. The cells were exposed to free AmB or AmB-loaded MCPs at an equivalent concentration of 50 *µ*g/mL of AmB and incubated for 24 h. Afterward, the cells were washed three times with PBS and incubated with acridine orange (AO) solution (1 *µ*g/mL, 2 mL) for 5 min at room temperature. After incubation with AO, the cells were washed twice with PBS and the glass cover-slips were mounted on a microscope slide using a spacer. Cells without any treatment were used as controls. The cells were imaged using an Olympus FluoView FV 1000 confocal microscope.

### 2.10. Statistical Analysis

Statistical analyses were conducted using Student's *t*-test (two-tailed distribution, two samples with unequal variances). Differences were considered significant at *P* < 0.05.

## 3. Results and Discussion

### 3.1. Characterization of AmB-Loaded MCPs

**Figure 1:**
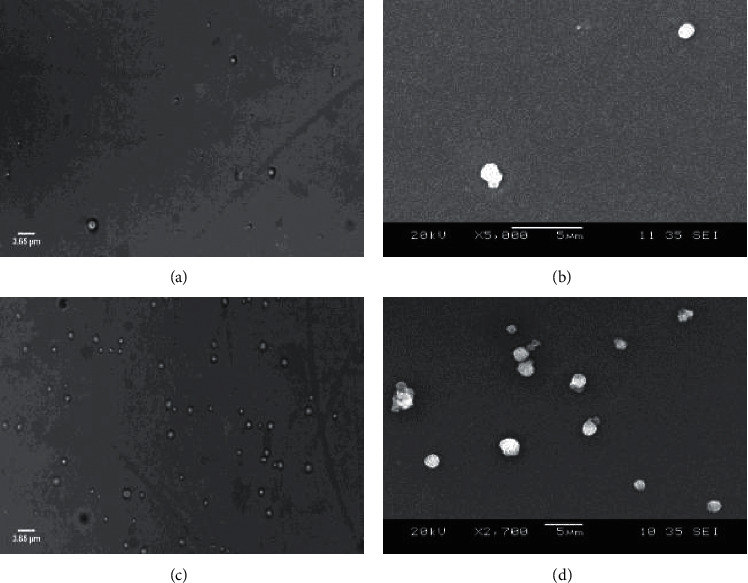
Particle size and morphology of alginate-based microparticles. Optical microscope (a) and SEM (b) images of AmB-loaded MCPs. Optical microscope (c) and SEM (d) images of blank microparticles (MCPs).

**Figure 2:**
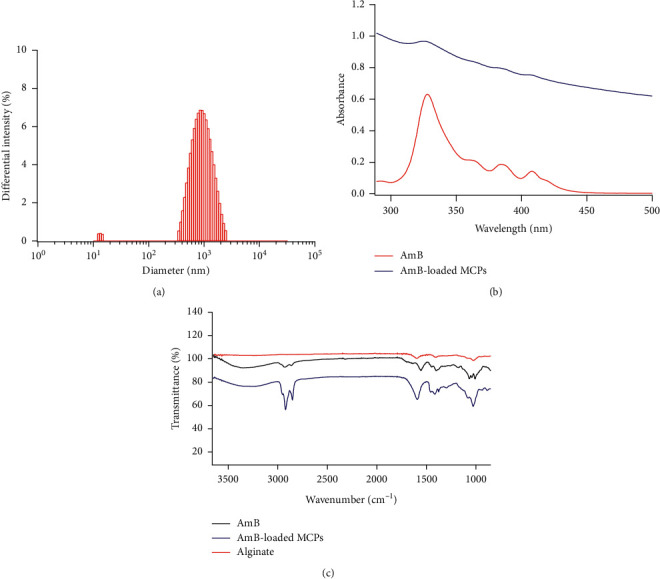
AmB-loaded MCPs characterization. (a) Hydrodynamic diameter of AmB-loaded MCPs. (b) Absorption spectra of MCPs-AmB and free AmB. (c) FTIR spectra of AmB-loaded MCPs, free AmB, and alginate.

In this work, an alginate-based microsystem for the delivery of the clinically relevant antifungal antibiotic AmB was fabricated using an emulsification method followed by cross-linking with calcium chloride [45]. The resulting AmB-loaded MCPs had a diameter of 1.3 ± 0.3 *μ*m according to SEM and optical microscope (Figure 1). The hydrodynamic diameter was 1.6 ± 0.4 *μ*m as determined by DLS (Figure 2(a)). The *ζ*-potential of the microsystem was −40.8 ± 2.2 mV. These results demonstrated that the synthesized AmB-loaded MCPs were not aggregated in solution as shown by the similarities in size obtained by DLS and SEM images (no statistically significant difference was observed between sizes obtained by SEM and DLS). This may be attributed to the large negative value of *ζ*-potential that prevents aggregation due to electrostatic repulsion of individual microparticles [55, 56]. Blank microparticles (MCPs) showed a diameter of 1.6 ± 0.3 *μ*m according to SEM and optical microscope. This indicates that the incorporation of AmB did not induce an increase in the diameter of the microplatform.

The FT-IR spectrum of AmB-loaded MCPs showed the characteristic absorption bands of AmB.  Figure 2(c) shows the stretching vibrations at 2923 cm^−1^ and 3349 cm^−1^ corresponding to CH_2_ and CH_3_ groups and OH, respectively [57, 58]. On the other hand, the absorption spectra of AmB-loaded MCPs showed the characteristic absorption peaks of AmB confirming the successful encapsulation of AmB into the alginate microparticles while remaining stable and intact (Figure 2(b)). The absorption spectra provide additional information about the aggregation state of AmB [42, 47]. Aggregates are responsible for inducing toxicity in mammalian cells and the ratio of absorbance of the first and fourth peak in the absorption spectra provides information regarding the extent of aggregation of AmB (a value less than 1 is characteristic of the monomeric form, while a value higher than 2 corresponds to the aggregated form) [47]. The ratio of the first and fourth peak for the AmB-loaded MCPs was 1.3 indicating that this vehicle may induce low toxicity.

The amount of AmB loaded on the microparticles was 6.11 ± 0.06 *µ*g of AmB/mg of microparticles and the percentage of encapsulation was 15.8% as determined by UV-Vis spectroscopy.

### 3.2. *In Vitro* Release Profile

**Figure 3:**
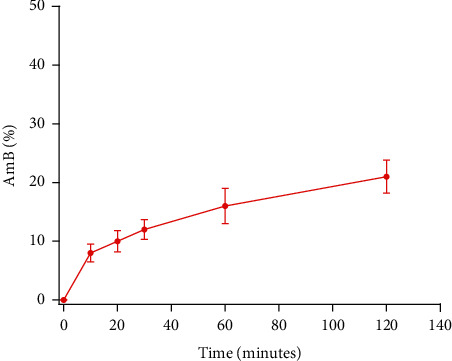
Release profile of AmB-loaded MCPs. Error bars represent standard deviation of three independent experiments.

An attractive aspect of polymeric-based vehicles is their ability to promote sustained release of drugs at concentrations capable of inducing a therapeutic effect. To evaluate the release of AmB from the alginate-based vehicle synthesized in this work, an *in vitro* drug release assay was performed. The results demonstrated that AmB was released from the alginate-based vehicle as shown in Figure 3. A burst of AmB released from the alginate vehicle was observed at the initial stage, followed by a slower release phase. During the first 60 minutes, 15% of the drug was released, and at 120 minutes 21% of the drug was released. This release behavior is similar to those reported in the literature [36]. An interesting aspect observed in the release profile is that the amount of drug released from our platform is higher than other alginate-based vehicles reported in the literature that have shown lower *in vitro* antifungal toxicity when compared to the free drug. For example, Ravichandran and Jayakrishnan encapsulated alginate-AmB conjugates into gels discs of gelatin cross-linked with oxidized alginate. The authors reported that the release from the gel discs was approximately 15–20% in 2 days and the antifungal efficacy against *Candida albicans* yeast was less than that of the free drug [42]. On the other hand, De Castro et al. fabricated alginate nanoparticles containing miltefosine. The results demonstrated that the *in vitro* effectiveness of the platform toward *Candida* spp. was lower than the free drug. The authors established that these results were correlated to the slow release of the free drug from alginate nanoparticles which was only 7.55% in 24 h [36].

Because one of the main concerns of current AmB formulations is the requirement for repeated intravenous injections for successful treatment resulting in high costs, it is important to develop formulations that promote a sustained release of drugs at concentrations that will induce a therapeutic effect similar to or higher than the free drug. Based on the release profile, we anticipate that our platform will induce an *in vitro* antifungal activity toward *Candida albicans*.

### 3.3. Evaluation of Activity of AmB-Loaded MCPs

**Figure 4:**
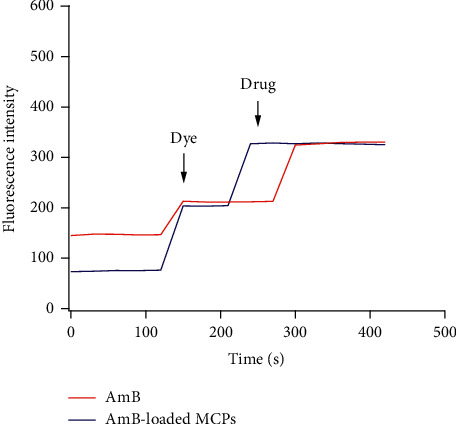
Membrane depolarization of *Candida albicans* cells by AmB-MCPs and free AmB using DiBAC_4_(3) dye.

The mechanism of action of AmB is based on the binding of the hydrophobic moiety of the molecule to the ergosterol found in the fungal cell membrane, leading to cell membrane damage and subsequent death of the microorganism [20]. Membrane depolarization studies were performed to evaluate the ability of the AmB encapsulated into the alginate microparticles to disrupt the cellular membrane of *Candida albicans* cells. To carry out this experiment, the anionic fluorescent probe DiBAC_4_(3) which only fluoresces in the microenvironment of the membrane was used. Normal cells exclude the dye because they have a negative internal charge. However, exposure to AmB induces membrane depolarization, which allows the dye to enter, bind to lipid-rich intracellular components, and fluoresce [48, 49, 59]. Thus, an increase in fluorescence after exposure to AmB indicates its ability to disrupt the cellular membrane. The results demonstrated that the microvehicle developed was able to disrupt the cellular membrane potential at the same level as free AmB (Figure 4). The first 120 seconds of Figure 4 shows a baseline corresponding to cells suspended in saline water. After addition of the fluorescent probe, an increase in intensity was observed due to the presence of the fluorescent compound; the fluorescence was measured until a stable signal intensity was achieved. After adding AmB or AmB-loaded MCPs, a significant increase in fluorescence was observed, suggesting the induction of membrane depolarization. This indicated that the activity of AmB was not compromised during the encapsulation process. In addition, the AmB released from the alginate-based vehicle may induce a therapeutic effect toward *Candida albicans* yeast at the same level as the free drug which in turn may result in avoiding the requirement for repeated intravenous injections for successful treatment.

### 3.4. Antifungal Efficacy of AmB-Loaded MCPs toward *Candida albicans* Yeasts

**Figure 5:**
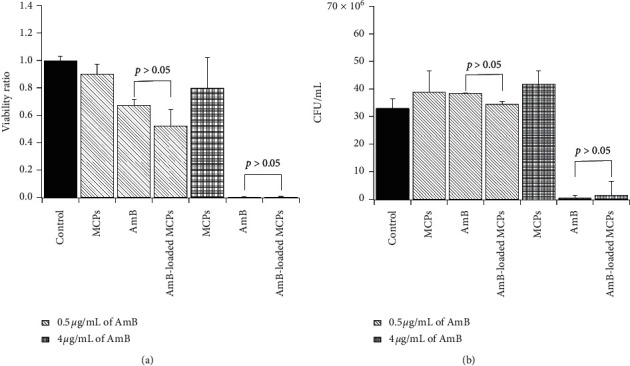
Cell viability: (a) viability ratio and (b) CFU/mL of *Candida albicans* cells treated with free AmB or AmB-loaded MCPs for 48 hours. The amount of AmB-loaded MCPs added corresponded to an equivalent amount of free AmB. MCPs represent blank microparticles at the concentration used for AmB-loaded MCPs. Error bars represent the standard error of two independent experiments.

It has been reported that *Candida albicans* is responsible for over 50% of all cases of invasive candidiasis [6, 7]. Therefore, *Candida albicans* cells were used to evaluate the antifungal effectiveness of the microplatform developed in this work. Cells were exposed to two concentrations of AmB (0.5 *μ*g/mL or 4 *μ*g/mL) for 48 hours at 35°C and the viability ratio and CFU/mL were determined using the cell counting method and plate count method, respectively. These concentrations were chosen in accordance to clinical concentrations reported in the literature [50, 51]. The cytotoxicity of blank MCPs at the corresponding concentration used for 0.5 *μ*g/mL and 4 *μ*g/mL was also evaluated. The results demonstrated that the efficacy of AmB-loaded MCPs is similar to that of free AmB (Figure 5). No statistically significant difference in viability ratio or CFU/mL was observed between cells treated with free AmB and cells treated with AmB-loaded MCPs at 0.5 *μ*g/mL or 4 *μ*g/mL. MCPs did not induce any significant cytotoxicity on *Candida albicans* cells at the concentration used in this work. This result confirmed that the decrease on cell viability is exclusively related to the AmB released from the alginate microparticles. The effectiveness of the platform developed in this work using the emulsification/external gelation method is higher when compared to other alginate-based platforms reported in the literature. For example, the *in vitro* antifungal activity of alginate-based nanoparticles containing miltefosine against *Candida* spp. was lower than that of the free drug [36]. A similar behavior was observed for AmB conjugates that were obtained by reacting AmB with sodium alginate via imine and amine linkages [42]. This high antifungal efficacy may be attributed to the fabrication procedure. It is known that the production methods of alginate nano- or microparticles have a significant impact on tuning the (bio)pharmaceutical performance [44]. Based on these results, this platform may not require repeated intravenous injections which is one of the limitations of commercial lipid formulations.

Additionally, several studies have reported that the utilization of carrier systems, such as liposomes, nanoparticles, and micelles, has reduced the main side effects of AmB [3, 26, 60, 61]. Thus, the use of the alginate-based drug delivery system could decrease significantly the side effects of this drug while inducing a therapeutic effect similar to the free AmB.

### 3.5. In Vitro Cytotoxicity of AmB-Loaded MCPs in Lung Cells

**Figure 6:**
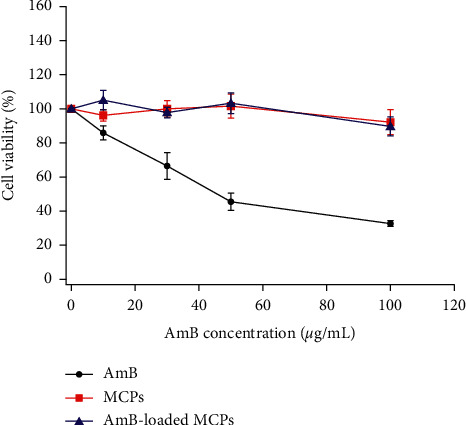
Cell viability of human lung cells treated with free AmB or AmB-loaded MCPs for 48 hours. The amount of AmB-loaded MCPs added corresponded to an equivalent amount of free AmB. MCPs represent blank microparticles at the concentration used for AmB-loaded MCPs. Error bars represent the standard error of four independent experiments.

Nephrotoxicity has been the most studied side effect induced by polymeric-based drug delivery systems containing AmB. The majority of the studies related to the fabrication of these vehicles have focused on evaluating their *in vitro* toxicity in human kidney cells, demonstrating their ability to significantly decrease the main side effects of AmB [25, 26, 42, 52, 53]. Nevertheless, the toxicity caused by polymeric formulations in lung cells has not been a focus of study. It has been reported that although the encapsulation of AmB into drug delivery systems (lipid formulations) decreased the main side effects of this drug; these formulations induced acute pulmonary toxicity [19, 23, 27–30]. On the other hand, Sangeetha et al. reported that encapsulating AmB into alginate/poly-L-lysine nanoparticles impacted the biodistribution of the drug, inducing high accumulation of AmB in the lungs while reducing it in the kidneys [43]. Thus, it is necessary to study the *in vitro* toxicity of AmB-loaded MCPs in lung cells which is the focus of our study. To this, human lung cells (H-460) were exposed to various concentrations of AmB (0–100 *μ*g/mL) for 48 h. The amount of AmB-loaded MCPs and MCPs used corresponded to an equivalent amount of free AmB and the concentration used for AmB-loaded MCPs, respectively. After this period of time, the cell viability was determined using the CellTiter 96® AQueous assay. The results demonstrated that free AmB induced toxicity in a dose-dependent manner with an IC50 of 50 *μ*g/mL (Figure 6). This is consistent with previous reports which established that high concentrations of AmB induced lung cell injury [29, 62]. On the other hand, blank microparticles (MCPs) and AmB-loaded MCPs did not induce lung cell toxicity in the range assessed. These results demonstrated that AmB-loaded MCPs were able to induce antifungal toxicity against *Candida albicans* yeast cells at the same level of free AmB with minimal toxicity in lung cells, which has been one of the main concerns when AmB is encapsulated into lipid-based drug delivery systems. Thus, this microplatform may not require several infusions for a successful treatment while reducing the pulmonary toxic effect induced by current commercial formulations.

### 3.6. Cell Membrane Injury

**Figure 7:**
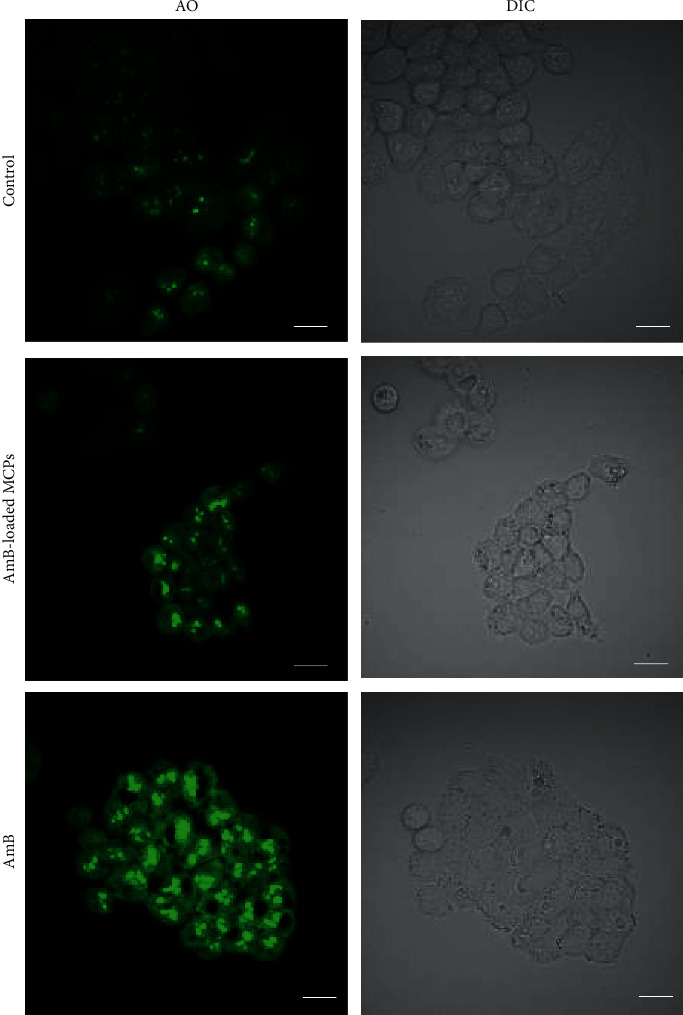
Acridine orange incorporation into lung cells treated with free AmB or AmB-loaded MCPs. Scale bar represents 20 *μ*m.

Although it is not clear why current AmB lipid formulations induce pulmonary toxicity, studies have reported that AmB directly injures cells in a dose- and time-dependent manner [29]. The injury could be caused by the formation of pores in cholesterol containing membranes [63]. Because it has been reported that alginate-based vehicles can accumulate at high concentrations in the lungs [43], qualitative diffusion analysis of acridine orange (AO) was performed in order to confirm that the vehicle developed in this work does not induce significant cell membrane damage. AO diffuses passively through the cellular membrane and it has been used to evaluate qualitatively membrane perturbation or damage [64]. If pores are formed on the cell membrane, the intracellular accumulation of AO will increase. Thus, an increase in the fluorescence intensity will be observed as it is directly proportional to the amount of dye that has entered into the cell. Our results demonstrated that, after exposing lung cells to AmB at 50 *μ*g/ml for 24 hours, the free drug had the ability to significantly increase the incorporation of AO into the cells when compared to untreated cells (Figure 7). Cells treated with AmB-loaded MCPs showed a slight increase in AO fluorescence when compared to the control. These results demonstrated that the free drug was able to significantly enhance the rate of passive diffusion of AO across the membrane, presumably due to severe cell membrane damage. On the other hand, the cell membrane damage caused by the alginate-based vehicle was minimal. It has been reported that the severity of the cell membrane damage is related to cell survival [65]. Cells have the ability to repair the cell membrane when minor damage occurs; nevertheless, when the damage is beyond their repair capacity, cell death will be induced [65]. Therefore, the cell damage caused by the alginate vehicle seems to be minor allowing the cell to repair the membrane. This had a positive impact on the cell viability as demonstrated by the toxicity assay. Taken together, the toxicity assay results and cell membrane injury studies indicate that the encapsulation of AmB into alginate microparticles using the emulsification/external gelation method may induce minimal pulmonary toxicity.

## 4. Conclusions

Our work demonstrated that AmB-loaded MCPs were effective in disrupting the cellular membrane potential and reducing the cell viability of *Candida albicans* cells at the same level as free AmB with minimal lung cell toxicity and cell membrane injury. These results indicate that the activity of the antifungal was not compromised during the fabrication process and the microplatform may not require several infusions for a successful treatment while reducing the pulmonary toxic effect induced by current commercial lipid formulations.

Although the antifungal toxicity induced by the free drug and AmB-loaded MCPs is statistically the same, one of the main side effects induced by current AmB-lipid formulations (pulmonary toxicity) may be reduced by encapsulating AmB into alginate microparticles using the emulsification/external gelation method.

Additionally, this microvehicle can be modified to provide targeting abilities to further impact the biodistribution of drugs and allow better selectivity. Future work will be focused on modifying the platform with targeting molecules to render target-specific properties toward *Candida albicans* cells and evaluate its effectiveness and impact on pulmonary toxicity. Overall, our results provide convincing evidence of the effectiveness of the platform developed in this work toward *Candida albicans* cells and its ability to likely reduce pulmonary toxicity.

## Data Availability

All data included in our manuscript are available.
